# Optic nerve sheath diameter/eyeball transverse diameter ratio by ultrasound in prediction of increased intracranial pressure in children with viral encephalitis

**DOI:** 10.3389/fped.2024.1485107

**Published:** 2025-01-07

**Authors:** Chun Zhao, Peng-Cheng Sun, Ke-Jie Fang, Hui-Hui Fu, Li-Feng Wei, Yin-Yun Miao, Xin-Xin Guo, Xiao-Ling Weng

**Affiliations:** ^1^ Department of Pediatrics, The Affiliated Yangming Hospital of Ningbo University, Yuyao People’s Hospital, Yuyao Branch of the Second Affiliated Hospital of Zhejiang University School of Medicine, Ningbo, China; ^2^ Department of Ultrasound, The Affiliated Yangming Hospital of Ningbo University, Yuyao People’s Hospital, Yuyao Branch of the Second Affiliated Hospital of Zhejiang University School of Medicine, Ningbo, China; ^3^ Department of Medical Imaging, Ditang Central Health Center, Yuyao People’s Hospital Medical Community, Ningbo, China

**Keywords:** pediatrics, optic nerve sheath diameter, ONSD/ETD, increased intracranial pressure, viral encephalitis, ultrasound

## Abstract

**Introduction:**

Increased intracranial pressure (ICP) is common with viral encephalitis in children which is associated with complications and prognosis. The optic nerve sheath diameter (ONSD) is a new indicator for the assessment of intracranial pressure using ultrasound, CT scan and MRI imaging. Given the influence of physical development on ONSD size in children, we expect more accurate assessment of intracranial pressure with ONSD/ETD (eyeball transverse diameter) ratio by ultrasound. The aim of the study is to determine the performance of the ONSD/ETD ratio measurement to predict ICP occurring in children with viral encephalitis and evaluate the therapeutic effect.

**Methods:**

Children with viral encephalitis from May 2022 to June 2024 were recruited in this study. The initial ONSD/ETD ratio measurement by ultrasound were completed before lumbar puncture. Children were divided into the increased ICP group and the normal ICP group based on whether the ICP was over 200 mmH_2_O measured by lumbar puncture. The ultrasound was repeated on the 3rd and 7th day of treatment.

**Results:**

The ONSD/ETD ratios measured in the two groups before treatment were 0.231 ± 0.019 and 0.182 ± 0.012, respectively (*p* < 0.01). The ONSD/ETD ratio on the 3rd day of treatment in the increased ICP group was significantly lower than the data before treatment (*p* < 0.01). The data on the 7th day of treatment in increased ICP group was significantly lower than the data before treatment (*p* < 0.01), but not statistically significant compared to the data on the 3rd day of treatment (*p* = 0.650). The ROC curve demonstrated an AUC for ONSD/ETD ratio in predicting the occurrence of increased ICP in children with viral encephalitis was 0.974 [95% confidence interval (CI): 0.939–1.000, *p* < 0.01], with a sensitivity of 95.1% and specificity of 93.3% at a cut-off value of 0.198.

**Conclusion:**

Our study shows that ONSD/ETD can be used as an easy reference tool for evaluating ICP in children with viral encephalitis which can reflect the therapeutic effect.

## Introduction

Viral encephalitis is a common disease in children's neurology. The clinical symptoms just like headache, vomiting and hyperspasmia are related to ICP. In severe cases, increased ICP can lead to cerebral hernia and consciousness disorder. Accurately monitoring changes in intracranial pressure, early identification of moderate to severe intracranial pressure elevation, and timely appropriate intervention are of great significance to improve the prognosis of brain function and reduce mortality in children.

The optic sheath is a continuation of the dura mater. When increased ICP is transmitted to the cerebrospinal fluid (CSF) inside the optic nerve sheath, the expansion of the sheath causes swelling of the optic nerve sheath and an increase in the gap inside the optic nerve sheath, resulting in an increase in the ONSD (1). ONSD become an indirect marker of changes in ICP due to the connectivity of CSF (2). The study found that there was a clear correlation between the increase in ONSD and the increase in ICP (3). US and MRI provide measurements of ONSD that are well-correlated and sensitive markers for increased ICP (4). The advantages of ultrasound lie in its convenience, speed, reduced cost, non-invasive modality, repeatability, lack of side effects and ability to perform bedside examinations. Many studies have showed that ultrasound measurement of ONSD can serve as a marker for increased ICP (2, 5–21). Different ONSD cut-off values are regardless of sex and age for the assessment of increased ICP in traumatic brain injury adults (22). However, the size of a child's eyeballs and optic nerve will increase with age. There was a study using ultrasound in fetuses which found an ONSD increase from 1.2 mm at 23 weeks to 2.6 mm at 36 weeks (23). Some researchers divided the healthy children after birth into four groups according to the age group: 1 month old to 2 years old, 2 years old to 4 years old, 4 years old to 10 years old, and 10 years old to 18 years old. The results showed that ONSD and ETD increased with age (24). It can be seen that ONSD and ETD measurements increase with age, body size and organ growth. Therefore, there is no consensus on the normal value of ONSD in children. Several studies have measured the optic nerve on CT and MRI in children and found no statistical differences in the ONSD/ETD ratio by age and sex (24, 25).

We expect ONSD/ETD ratio to evaluate ICP more accurately than ONSD alone in children, as it removes the influence of factors such as age and body size. The aim of this study was to evaluate the predictive value of ONSD/ETD ratio in relation to increased intracranial pressure in children with viral encephalitis.

## Methods

### Patients

This study recruited children who were diagnosed with viral encephalitis from May 2022 to June 2024 in the Department of Pediatrics, Yuyao Branch of the Second Affiliated Hospital of Zhejiang University. The diagnosis of viral encephalitis was based on the criteria of the International Encephalitis Collaboration Group. The study has been approved by the Ethics Committee of the Yuyao People's Hospital. All children participated voluntarily and had informed consent signed by their legal guardians. The exclusion criteria were as follows: (1) >16 years old; (2) anterior fontanel is not closed; (3) suffered from eye disease, open head trauma or intracranial tumour; (4) history of medication affecting intracranial pressure; (5) other conditions that do not cooperate with the inspection. Sex, age, body mass index (BMI), blood pressure, symptoms, signs and disease duration were be recorded.

### Ultrasound

The ultrasound examinations were conducted by a clinical physician who had received professional theoretical and practical training in critical ultrasound, which proved that he can perform measurements. All examinations were conducted simultaneously under the supervision of an ultrasound physician skilled in neurovascular examination. The measurements were obtained by a Mindray M9 ultrasound device with a high-frequency linear transducer (frequency 7.5 MHz). The patient was placed in a supine position with the head between 20° and 30°, the protocol was started with the right eye. The ultrasound gel was applied to the surface of the patient's closed upper eyelid, and the probe was gently placed on the eyelid without pressure. The probe was placed on the transverse section of the eyeball, and the optimal plane was scanned from the frontal end to the nasal end to fully demonstrate the path of optic nerve entering the eyeball. The maximum outer diameter of optic nerve sheath at 3 mm behind the ball and the maximum transverse diameter of eyeball in this plane were measured respectively, namely ONSD and ETD. The procedure was repeated three times on the transverse section; then, the probe was positioned on the vertical section, and the measurement of ONSD was again repeated three times. The entire ultrasound assessment was performed for the contralateral eye later. To reduce measurement errors, the final ONSD/ETD values were averaged. The mean values of right and left eyes were used for statistical analysis. Ultrasound examinations were performed before, 3 days and 7 days after clinical treatment.

### Lumbar puncture

The lumbar puncture was performed within 30 min after the ultrasound examination by a senior physician. The children were placed in the left lateral decubitus position with the neck and knees fixed by the assistant. L3–L4 or L4–L5 was selected as puncture point, and after disinfection, wrapping, and local anesthesia, a puncture needle was inserted from the puncture point along the anesthesia canal until a breakthrough is felt. After seeing the outflow of the cerebrospinal fluid, connecting the pressure-measuring tube, and making the children straighten their legs slowly, the ICP was read after the water column stabilized. All children were divided into the increased ICP group and the normal ICP group, where ICP >200 mmH_2_O was considered to be increased.

### Statistical analysis

Statistical analyses were performed using IBM SPSS 19.0 software. Normally distributed continuous variable were expressed as mean ± standard deviation (SD) and non-normally distributed continuous variables were represented by median and interquartile range (IQR). One-way ANOVA analyses of variance were applied to evaluate the differences in ONSD/ETD before and after treatment. The differences between each time point were analyzed using Student's *t*-tests when the overall difference has statistical significance. ROC curve was used to evaluate the accuracy of visual ONSD/ETD ratio in increased ICP assessment, and the area under the curve and 95% confidence interval (CI), threshold, sensitivity and specificity were calculated. Statistical significance was set at *p* < 0.05.

## Results

56 children with viral encephalitis were included for the statistical analysis. Patients' demographic and clinical characteristics are described in Table 1. The average age of children was 7.98 ± 2.88 years old in this study. Male accounted for 66.07% and female 33.93%. BMI ranged from 12.54 (kg/m^2^) to 20.76 (kg/m^2^). The analysis of systolic and diastolic blood pressure data indicated that there was no hypertension or hypotension in this study. All children had one or more symptoms and signs associated with increased ICP, 85.71% had headache, 57.14% had vomiting and 17.86% had convulsion. 5.36% of patients exhibited a positive meningeal irritation sign and 17.86% showed a positive Babinski sign.

**Table 1:** This table contains information on children with viral encephalitis demographic and clinical data.

Group	Total	Increased ICP	Normal ICP
Number	56	41	15
Age (years), mean (SD)	7.98 ± 2.88	8.13 ± 3.11	7.55 ± 2.10
Sex			
Male	37 (66.07%)	28 (68.29%)	9 (60%)
Female	19 (33.93%)	13 (31.71%)	6 (40%)
BMI (kg/m^2^), median [IQR (25th–75th)]	14.79 (13.75, 16.74)	14.87 (13.79, 17.16)	14.71 (13.72, 15.29)
Blood pressure (mm/Hg), median [IQR (25th–75th)]			
SBP	106 (100, 110)	106 (98.5, 110)	107 (100, 115)
DBP	64 (58, 72)	65 (61, 71)	64 (55, 76)
Signs and symptoms			
Fever	47 (83.93%)	34 (82.93%)	13 (86.67%)
Headache	48 (85.71%)	40 (97.56%)	8 (53.33%)
Vomiting	32 (57.14%)	29 (70.73%)	3 (20%)
Convulsion	10 (17.86%)	5 (12.20%)	5 (33.3%)
Personality changes or Psychobehavioral abnormality	8 (14.29%)	3 (7.32%)	5 (33.3%)
Meningeal irritation sign (+)	3 (5.36%)	3 (7.32%)	0 (0)
Babinski sign (+)	10 (17.86%)	7 (17.07%)	3 (20%)

**Figure 1:**
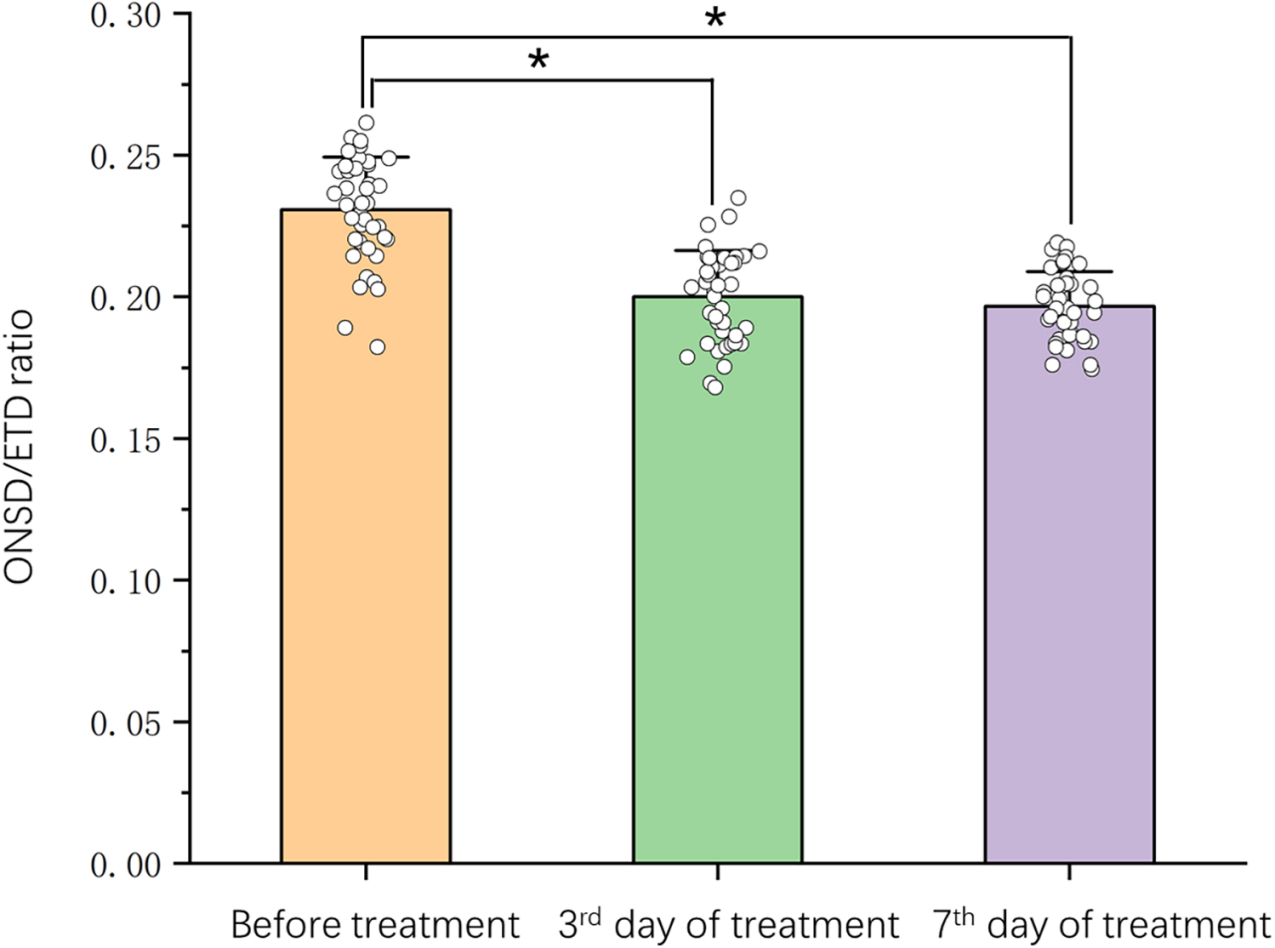
Changes of ONSD/ETD ratio (mean ± SD) before and after treatment in children with viral encephalitis. *p < 0.05 different between groups. Scatter plot comparing time course (on *x*-axis) and ONSD/ETD ratio (on *y*-axis).

The ONSD/ETD ratios measured in increased ICP group and normal ICP group before treatment were 0.231 ± 0.019 and 0.182 ± 0.012, respectively (*p* < 0.01). After treatment such as reducing intracranial pressure with mannitol, the ONSD/ETD ratio on the 3rd day of treatment in increased ICP group was 0.200 ± 0.016 which was significantly lower than the data before treatment (*p* < 0.01). The patients' clinical symptoms had improved in this stage. The last ONSD/ETD measured on the 7th day of treatment in increased ICP group was 0.197 ± 0.012, which was significantly lower than the data before treatment (*p* < 0.01), but not statistically significant compared to the data on the 3rd day of treatment (*p* = 0.650). The parameters are detailed in Figure 1. There was no significant difference in ONSD/ETD before and after treatment in the normal ICP group (*F* = 0.324, *p* = 0.725).

**Figure 2:**
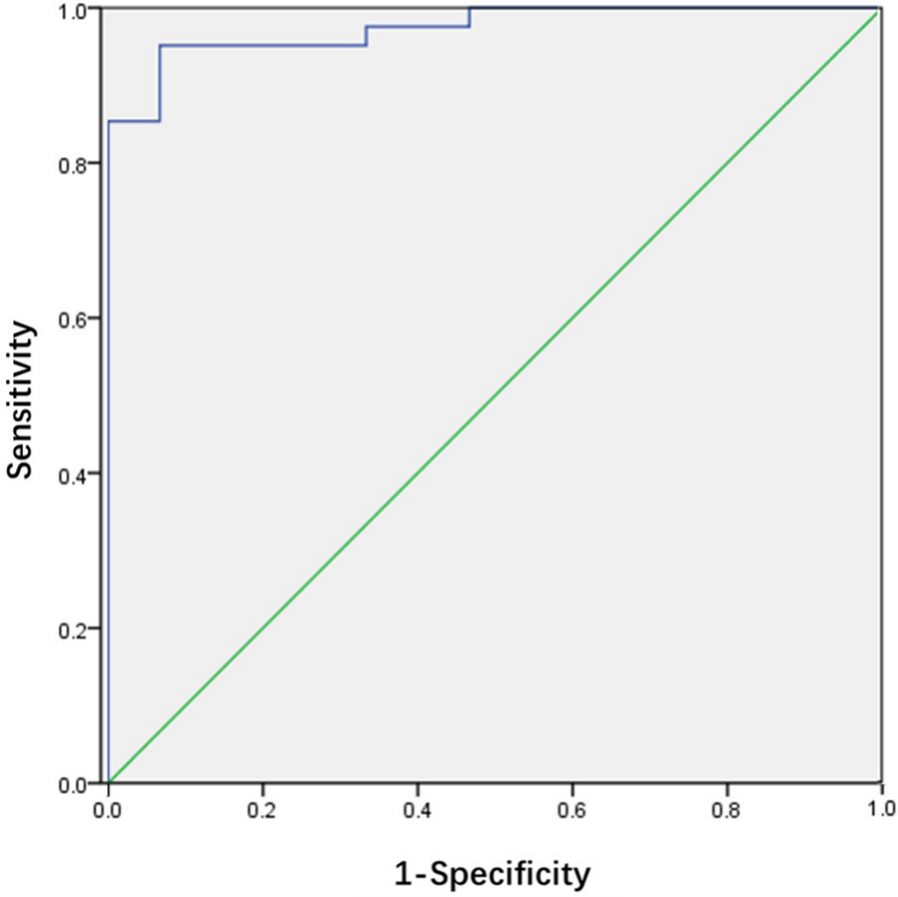
Receiver operating characteristic curve for the ONSD/ETD ratio. The area under the curve was 0.974 (95% CI: 0.939–1.000).

The efficiency of ONSD/ETD ratio in predicting the occurrence of increased ICP in children with viral encephalitis is shown in Figure 2. The ROC curve demonstrated an AUC for ONSD/ETD ratio in predicting the occurrence of increased ICP in children with viral encephalitis was 0.974 [95% confidence interval (CI): 0.939–1.000, *p* < 0.01], with a sensitivity of 95.1% and specificity of 93.3% at a cut-off value of 0.198.

## Discussion

Most viral encephalitis in children causes increased ICP and the increased ICP group accounted for 73.21% of the total as shown in this study. A mild elevation in ICP can cause symptoms like headache and vomiting. As the ICP continues to rise, it can lead to papilledema, blurred vision, and seizures. Further exacerbation may result in consciousness disorders and cerebral herniation. All of the children in the increased ICP group had one or more symptoms of increased ICP, while only 20% of the normal ICP group had headaches, 13.3% had vomiting, and 33.3% had convulsions. Based on clinical experience, we believe that the above symptoms in the normal ICP group may be related to the pathogenicity of infection with the virus, such as rotavirus easily causing vomiting and convulsions caused by electrolyte disturbance in gastroenteritis. In addition, fever can also lead to headaches and high fever convulsions. It can be seen from this that even though some children with viral encephalitis may have individual symptoms of increased ICP, the actual ICP has not truly increased. The measurement and evaluation of ICP at this time is of great significance for subsequent medication and efficacy evaluation.

The gold standard for intracranial pressure detection is invasive direct measurement, including traditional lumbar puncture and more direct and precise intracranial probe implantation. These invasive methods are more suitable for patients with craniocerebral trauma or chronic intracranial diseases, and are not suitable for children with acute viral encephalitis because the disadvantages outweigh the benefits. Ultrasound has emerged as a new option for dynamic assessment of ICP because it is non-invasive, non-radiative and reproducible. ONSD was thought to be used to determine the presence of cranial hypertension in the studies of adults originally. The measurement method of ONSD by ultrasound is relatively simple and easy to master and operate. The clinician has the ability to operate independently in the absence of an ultrasound specialist. Because the thickness of the optic nerve is proportional to the size of the eyeball and is associated with age, ONSD/ETD ratio is recommended to evaluate ICP instead of ONSD in order to reduce the impact of this factor (26–28). Our data support this conclusion. Several researches have suggested that changes in the ONSD/ETD ratio on the CT scan are more effective than ONSD in detecting increased ICP (28–30). However, it is worth noting that two-dimensional ultrasound is affected by the measurement Angle, and the widest transverse eyeball diameter measured in the same image measuring ONSD is ETD. But this measurement will not be the same as the ETD diameter used in tomographic measurements. Tomography can show the full picture of the eyeball and even reconstruct a three-dimensional image, so the maximum transverse diameter of the eyeball can be measured most accurately. Ultrasound cannot do this, and the ultrasonographic image obtained does not actually measure the widest diameter of the eyeball. It is understood that the definition of ONSD/ETD ratio in CT and ultrasonic measurements is slightly different. Still, Nihan et al. found that ONSD/ETD on CT scan was quite sensitive to detect increased ICP in pediatric head trauma and there was a significant correlation between measurements calculated by ultrasound and CT scans (31). More research confirms that the ONSD/ETD ratio tested by ultrasound may be a reliable indicator for predicting increased ICP (32–34). Our study also supports the role of ONSD/ETD in determining increased ICP. Moreover,` the comparison before and after treatment was added in the research. The statistical difference also suggests that ONSD/ETD may be a suitable indicator for monitoring ICP, whose objective is to adjust the medication and treatment regimen in time according to the ICP.

Other researchers have sounded a different note which showed no correlations between increased ICP and ONSD, ETD or ONSD/ETD ratio on CT scan in neurotrauma patients (35). We wondered if this result might be related to the severity of the neurotrauma. The ONSD cannot expand indefinitely when nerve trauma reaches a certain level. Halil Onder's study found that the ONSD and ONSD/ETD ratio may be utilizable in the assessment of ICP in patients with idiopathic intracranial hypertension, but they cannot reach enough discriminant precision. Out study showed that there was no significant difference between the data of ONSD/ETD in the 3rd day and 7th day of treatment. But in terms of mean and median, the data of 7th day of treatment was lower than 3rd day. Our study did not uniform the severity of viral encephalitis, and the drug dosage was determined by the condition, with individual differences. Therefore, we believe that changes in ONSD/ETD are more pronounced during the first 3 days when symptoms improve significantly. In the later stage after significant relief of symptoms in increased ICP, as the drug dosage decreased, the changes in ONSD/ETD were not observable. All in all, more researchers and clinicians are needed to repeatedly test the true effectiveness of ONSD/ETD. The limitations of our study lied in its single-center and a small sample size, which was prone to bias. In the future, more in-depth research with multicenter large sample is needed.

## Limitations

This study had some limitations. In accordance with treatment guidelines and ethical requirements for viral encephalitis in children, invasive dynamic monitoring of intracranial pressure after treatment was not performed in this study. Thus, the most intuitive intracranial pressure data were lacking. In addition, it is not clear whether direct inflammatory stimulation of the optic nerve by encephalitis virus has an effect on the ONSD measurement data.

## Conclusion

ONSD/ETD ratio measured by ultrasound is an accessible tool to identify increased ICP in children with viral encephalitis. It is also an innovative and convenient way to dynamically monitor ICP changes during treatment. Further studies are required to reveal the benefits of ONSD/ETD ratio measurements by ultrasound in the course of increased ICP caused by viral encephalitis.

## Data Availability

The raw data supporting the conclusions of this article will be made available by the authors, without undue reservation.
